# Genome Sequencing of Four Strains of *Rickettsia prowazekii*, the Causative Agent of Epidemic Typhus, Including One Flying Squirrel Isolate

**DOI:** 10.1128/genomeA.00399-13

**Published:** 2013-06-27

**Authors:** Kimberly A. Bishop-Lilly, Hong Ge, Amy Butani, Brian Osborne, Kathleen Verratti, Vishwesh Mokashi, Niranjan Nagarajan, Mihai Pop, Timothy D. Read, Allen L. Richards

**Affiliations:** Naval Medical Research Center (NMRC)–Frederick, Fort Detrick, Maryland, USAa; Henry M. Jackson Foundation, Bethesda, Maryland, USAb; Naval Medical Research Center, Silver Spring, Maryland, USAc; BioTeam, Inc., Middleton, Massachusetts, USAd; University of Maryland, College Park, Maryland, USAe

## Abstract

*Rickettsia prowazekii* is a notable intracellular pathogen, the agent of epidemic typhus, and a potential biothreat agent. We present here whole-genome sequence data for four strains of *R. prowazekii*, including one from a flying squirrel.

## GENOME ANNOUNCEMENT

The genus *Rickettsia* includes numerous agents of mammalian disease. They are obligate intracellular bacteria and are regarded as the closest relatives to the ancestor of mitochondria (1). *Rickettsia prowazekii* is the causative agent of epidemic typhus, vectored primarily by the human body louse, and sylvatic epidemic typhus, which is associated in the United States with contact with the southern flying squirrel, *Glaucomys volans*. Although improved hygiene has mostly eradicated louse-borne epidemic typhus in the United States, there have been a number of sporadic cases of sylvatic epidemic typhus associated with flying squirrels (2–4).

Four genomes of *R. prowazekii* from three continents were sequenced using the 454 Titanium pyrosequencer. *R. prowazekii* strains Breinl, Madrid E, and Cairo were isolated from patients in Poland, Spain, and Egypt, respectively, while the flying squirrel strain, GvF12, was from the United States. Breinl and Cairo are virulent strains while Madrid E is avirulent. Madrid E was previously sequenced by another group of researchers but it was included in this project as an interesting control for lab-to-lab strain variation or sequencing platform-induced sequence variations. Coverage ranged from 39- to 145-fold average depth. The Naval Medical Research Center (NMRC) Madrid E and Breinl genomes were *de novo* assembled into 197 and 60 contigs, respectively; *in silico* gap closure was used to resolve each assembly into a single contig, and the process is described for the NMRC Madrid E genome in Nagarajan et al. (5). *De novo* assembly of the Cairo and GvF12 genomes resulted in 752 and 46 contigs, respectively. As expected, each of the genomes sequenced in this study is just over 1.1 Mb in size, with a fairly high percentage of noncoding regions, ~25 to 26%. Each genome was found to contain 33 tRNA genes and have a GC content of ~29%.

To date, there have been only two publically available genomes for flying squirrel isolates, which has limited conclusions that could be drawn regarding characteristics of strains that are isolated as part of the human-body louse cycle versus the flying squirrel-arthropod life cycle. Therefore, the genome of the flying squirrel isolate GvF12 was compared to the published flying squirrel strain GvV257 and GvF24 genomes and the previously published Madrid E genome, NCBI accession number AJ235269.1. Interestingly, the draft GvF12 genome was found to differ from the GvV257 and GvF24 genomes at 226 and 11 positions, respectively, whereas the GvF12 and Madrid E genomes were found to vary at 869 positions. By comparison, the Breinl and Madrid E genomes were found to differ at 292 positions. These preliminary data indicate that flying squirrel isolates may be more similar to each other than to human isolates. Further characterization of these and other isolates should aid our understanding of the biology of *R. prowazekii* in flying squirrels and the possible relationship to sporadic *R. prowazekii* infection of humans.

### Nucleotide accession numbers.

GvF12, Cairo 3, Madrid E, and Breinl genomes were deposited in GenBank under accession numbers APMN00000000, APMO00000000, CP004888, and CP004889, respectively. The versions described herein are the first versions.
